# Noninvasive brain stimulation for the treatment of auditory verbal hallucinations in schizophrenia: methods, effects and challenges

**DOI:** 10.3389/fnsys.2015.00131

**Published:** 2015-10-12

**Authors:** Katharina M. Kubera, Anja Barth, Dusan Hirjak, Philipp A. Thomann, Robert C. Wolf

**Affiliations:** ^1^Center for Psychosocial Medicine, Department of General Psychiatry, University of HeidelbergHeidelberg, Germany; ^2^Department of Psychiatry, Psychotherapy and Psychosomatics, Saarland UniversityHomburg, Germany

**Keywords:** transcranial magnetic stimulation, auditory verbal hallucinations, phenotypes, schizophrenia, brain stimulation, brain function

## Abstract

This mini-review focuses on noninvasive brain stimulation techniques as an augmentation method for the treatment of persistent auditory verbal hallucinations (AVH) in patients with schizophrenia. Paradigmatically, we place emphasis on transcranial magnetic stimulation (TMS). We specifically discuss rationales of stimulation and consider methodological questions together with issues of phenotypic diversity in individuals with drug-refractory and persistent AVH. Eventually, we provide a brief outlook for future investigations and treatment directions. Taken together, current evidence suggests TMS as a promising method in the treatment of AVH. Low-frequency stimulation of the superior temporal cortex (STC) may reduce symptom severity and frequency. Yet clinical effects are of relatively short duration and effect sizes appear to decrease over time along with publication of larger trials. Apart from considering other innovative stimulation techniques, such as transcranial Direct Current Stimulation (tDCS), and optimizing stimulation protocols, treatment of AVH using noninvasive brain stimulation will essentially rely on accurate identification of potential responders and non-responders for these treatment modalities. In this regard, future studies will need to consider distinct phenotypic presentations of AVH in patients with schizophrenia, together with the putative functional neurocircuitry underlying these phenotypes.

## Introduction

Auditory verbal hallucinations (AVH) are defined as auditive perceptions involving a verbal aspect in the absence of a provoking external stimulus (Aleman and de Haan, 1998). They represent a core symptom of schizophrenia and related spectrum disorders, but they also frequently occur in other psychiatric entities and in the non-psychiatric general population. In schizophrenia the term AVH comprises a multi-dimensional and heterogeneous group of symptoms that can be differentiated by certain phenomenological aspects such as subjective loudness, acoustic clarity, location and subjective reality. About 60–80% of patients affected by schizophrenia experience AVH (Aleman and de Haan, 1998; Hugdahl et al., 2008), such as conversing, commenting or imperative spoken speech in distinct voices. These symptoms, especially when the verbal content is experienced as negative, intrusive or persecutory, often induce high levels of distress and lead to significant psychosocial impairment. AVH are a highly relevant feature of schizophrenia that have attracted extensive clinical, phenomenological and neurobiological interest, yet treating these symptoms, especially in persons suffering from persistent AVH which do either not or not sufficiently respond to psychopharmacotherapy, is still a major clinical challenge. In approximately 25% of patients with schizophrenia, AVH are refractory to psychotropic drug treatment and can chronically persist (Shergill et al., 1998). Currently, there are no randomized controlled trials available which specifically investigated effects of psychopharmacotherapy (either monotherapy or combined drug regimes) on AVH severity reduction or full symptom remission. In a recent review (Sommer et al., 2012), data from the European First-Episode Schizophrenia Trial (EUFEST) was used to assess effects of five antipsychotic agents on AVH severity. Superiority of one treatment option against another was not confirmed for AVH severity (Sommer et al., 2012). Clinically, clozapine is still the drug of choice for patients with AVH who are resistant to two other antipsychotic agents. At present, no clinical trial has been published that specifically compares the efficacy of clozapine in comparison to other antipsychotic agents in the treatment of drug-resistant AVH.

## Transcranial Magnetic Stimulation

Given the need for effective treatment modalities, it is not surprising to see that over the past decade brain stimulation techniques have been increasingly used to ameliorate symptom burden in patients with schizophrenia suffering from persistent and mostly pharmacorefractory AVH. Among these approaches, a possible augmentation strategy for the treatment of psychiatric disorders (in particular catatonia and severe depression) is elctroconvulsive therapy (ECT). For patients with schizophrenia a meta-analysis of 10 double-blind RCT showed a significant effect for ECT (Tharyan and Adams, 2005), although none of the studies provided any specific details on AVH improvement (Sommer et al., 2012). In a recent review Nieuwdorp and colleagues summarized different stimulation methods including transcranial magnetic stimulation (TMS), ECT and transcranial Direct Current Stimulation (tDCS) in patients with medication-resistant psychosis (Nieuwdorp et al., 2015). The authors concluded that currently there is only weak evidence for stimulation techniques to relieve pharmacorefractory psychosis. Specifically considering AVH, further studies are needed to draw any strong conclusions about ECT as a treatment option for patients presenting with persistent AVH.

In the last decade, TMS has evolved into a therapeutic modality for several psychiatric and neurological symptoms. In particular, TMS is widely used to treat patients with major depression, obsessive-compulsive disorder (OCD) and specific symptoms of schizophrenia (AVH and negative symptoms) (Slotema et al., 2010). Its application as an adjunctive therapy is currently proposed by European specialists with evidence level C (Lefaucheur et al., 2014) taking into account that it is generally regarded as safe. We consider the application of TMS for treating individuals presenting with persistent AVH as paradigmatic. The use of TMS impressively illustrates a translational approach from basic neuroscience/neuroimaging to clinical treatment. However, it also illustrates fundamental methodological, neurobiological and phenomenological questions and challenges, which we will refer to in the following paragraphs.

### TMS: Putative Mechanisms of Action

TMS is a technique which allows a non-invasive stimulation of cortical neurons through the scalp. Originally, TMS was implemented as a neurophysiological tool for the study of the human motor system (Barker et al., 1985). Put simply, TMS uses a strong pulse of electrical current in a coil which is placed over the brain generating rapidly pulsating magnetic fields, which pass through the scalp, skull, and meninges, into the brain (Wassermann and Zimmermann, 2012). Thus, changing magnetic fields produce electrical impulses that stimulate superficial cortical neurons 2–3 cm below the device (Wassermann and Zimmermann, 2012). Modern devices can generate a rapid succession of pulses, called repetitive TMS (rTMS) by producing a relatively powerful magnetic field (about 1.5–3T), but only lasting very shortly (ms) (George and Aston-Jones, 2010). Frequencies of 1 Hz or lower are considered to be inhibitory, while frequencies of 5 Hz and higher are considered to be excitatory (Aleman, 2013). The specific topology of the induced electrical field in the brain is a source of uncertainty, because it is influenced by the complex shape and diverse conductivity of the cranial contents (Wassermann and Zimmermann, 2012), e.g., cerebrospinal fluid and foraminas in cranial bone. Long-term potentiation (LTP) and long-term depression (LTD) are believed to be key processes underlying long-term effects of rTMS (Chervyakov et al., 2015). *In vitro* experiments of hippocampal slice cultures suggest that rTMS can alter cortical excitability in terms of LTP of synaptic transmission inducing an increase in synaptic strength and postsynaptic AMPA receptor changes (Vlachos et al., 2012). At the level of functional connectivity, long-lasting enhancement is reflected by increased hippocampal-cortical network coupling after rTMS (Wang and Voss, 2015).

### How Effective is rTMS in the Treatment of AVH?

As a target region for rTMS in patients with AVH the superior temporal cortex (STC) is of special interest given converging multimodal imaging evidence suggesting a crucial role in AVH generation and perception (Allen et al., 2008; Waters et al., 2012). The rationale for stimulating this region is to inhibit cortical overactivity and potentially influence generative phenomena (i.e., AVH) which are thought to be closely associated with regionally increased cortical activity. Up to now, several randomized sham-controlled studies targeting the left temporoparietal cortex have been conducted and summarized in seven meta-analyses revealing effect sizes (Hedges’ “g”) ranging from 0.42 (i.e., a close to moderate effect) to 1.04 (regarded as high effect; Aleman et al., 2007; Tranulis et al., 2008; Freitas et al., 2009; Slotema et al., 2012, 2014). With the inclusion of the studies with larger patient samples, the mean weighted effect size of rTMS directed at the left temporoparietal area for AVH appears to decrease over time, although the effect is still significant (Slotema et al., 2012, 2014; Hoffman et al., 2013). Of note, Slotema and colleagues showed that the effect of rTMS was no longer significant at one month of follow-up revealing a mean weighted effect size of 0.40 (95% confidence interval = −0.23–0.102; Slotema et al., 2012). For a detailed description of the included studies, please see tables provided by Slotema and colleagues. Side effects were mild and the number of dropouts in the real TMS group was not significantly higher than in the sham group. Only few MRI studies investigated other regions than the left temporoparietal area as target regions for rTMS. Abnormal activation of the right hemisphere regions such as the inferior frontal gyrus and the postcentral gyrus is a frequently reported finding in patients who experience persistent AVH (Kuhn and Gallinat, 2012). Activation changes have been most consistently shown for areas of the prefrontal and temporal cortices (Allen et al., 2007; Sommer et al., 2008; Raij et al., 2009). Based on former findings in neuroimaging studies that both the right and the left temporal activation are associated with AVH (Shergill et al., 2000; Sommer et al., 2007) three studies directed rTMS at the right comparing with the left temporoparietal gyrus for the treatment of AVH (Lee et al., 2005; Jandl et al., 2006; Loo et al., 2010). According to these studies, no superior effects of right-sided stimulation (Slotema et al., 2014) were observed. Correspondingly, neither stimulation of Brocaś area nor its contralateral homologue was an effective target (Schonfeldt-Lecuona et al., 2004). Overall, these findings support the notion that deficient generation, monitoring and perception of inner speech rather than speech expression are disrupted functions in patients with persistent AVH (Shergill et al., 2000; Wolf et al., 2011). Abnormal STC function clearly plays a critical role in the expression of AVH, especially in those patients presenting with chronic and treatment-refractory symptoms. Recent studies showed that stimulation of this region with low-frequency rTMS may reduce the severity and frequency of AVH in schizophrenia patients, but the duration of the effect of rTMS may be less than one month (Slotema et al., 2012).

## Methodological Issues with TMS and the Challenge of Treating Phenotypic Diversity

As briefly discussed in the previous paragraph, it is noteworthy that therapeutic effects of rTMS in AVH patients are not long-lasting, and that along with publication of studies with larger patient populations, the effect size of rTMS over the left temporoparietal area has decreased over time (Slotema et al., 2012, 2014). Several studies published between 2004 and 2014 did not observe beneficial effects of rTMS in the treatment of persistent AVH (Schonfeldt-Lecuona et al., 2004; Slotema et al., 2011; Blumberger et al., 2012; Rosenberg et al., 2012; Bais et al., 2014). Several reasons may account for these phenomena. Two specific aspects of stimulation will be discussed, which may be superior to left sided STC intervention and which may also account for these variable results. Subsequently we will address the problem of the phenotypic diversity which is inherent to AVH both at the neural and phenomenological level.

### Is Bilateral Stimulation Superior?

It may be conceivable that bilateral could be superior over unilateral stimulation, especially given known dissociations of left- vs. right-hemispheric function. Up to now, however, only one study examined bilateral rTMS of the TPJ (Bais et al., 2014). The authors suggested that AVH frequency might be one of the most sufficient parameters to measure the responsiveness of left sided rTMS. In comparison, right-sided rTMS allows for a more complete management of AVH in terms of emotional and non-linguistic aspects which are suggested to originate in the right hemisphere. Contrary to their prediction, however, Bais and could not show any beneficial effect of bilateral rTMS in comparison to left sided rTMS and sham in improving AVH (Bais et al., 2014). Neurophysiological aspects such as transcallosal inhibition, and fewer rTMS impulses (50%) in a bilateral design (Thiel et al., 2006; Bais et al., 2014) might account for these negative results.

### Is STC stimulation alone sufficient?

The functional dominance of STC stimulation over other brain regions has been questioned by accumulating neuroimaging data acquired in patients with AVH. For instance, an association between AVH-severity and STC gray matter volume loss has been suggested by univariate voxel-based morphometry studies (Modinos et al., 2013). In contrast, using a multivariate statistical approach for structural data analysis, two distinct abnormal structural networks were recently identified in patients with persistent AVH, including a bilateral prefrontal system and a bilateral temporal/medial frontal network (Kubera et al., 2014). The latter structural network also differed between patients with persistent AVH compared to non-hallucinating patients (Kubera et al., 2014). It is possible that unilateral temporoparietal stimulation might not be sufficient to induce a relevant neuronal change in both networks, whose mutual interplay has still to be determined. Also, the relationship between structure and function still remains unresolved, e.g., in individuals with persistent AVH the impact of neural loss to neural network transmission, including effects in more remote neural networks, is unclear.

From a functional point of view, both “symptom capture” (i.e., inferring AVH-related brain activity from symptom occurrence) and “symptom interference” (i.e., inferring AVH-related brain dysfunction from paradigm-driven data) MRI studies have been conducted to investigate neural activation patterns in schizophrenia patients experiencing treatment-resistant AVH (Lawrie et al., 2002; Mechelli et al., 2007; Wolf et al., 2011). The vast majority of these studies focused on brain activity in speech-related pathways (Lavigne et al., 2015), according to the prevailing model of AVH suggesting a link between symptom generation and dysfunctional inner speech perception and monitoring (Hugdahl et al., 2008). From these studies, the left STC emerged as regions linked to AVH and in turn set the rationale for targeted stimulation. Yet the left temporal cortex, although a crucial neural node for hallucinatory symptom expression, is not the sole region which is thought to be involved in AVH generation and persistence. The prefrontal cortices have been frequently found to exhibit abnormal neural activity in patients with AVH, both at the level of regional function and at the level of functional connectivity (Kuhn and Gallinat, 2012; Alderson-Day et al., 2015). Although the processes subserved by abnormal prefrontal activity in patients experiencing AVH are not fully elucidated at present, several explanations have been put forward, such as deficient attentional and executive control over speech- and self-monitoring relevant brain regions. In addition, converging evidence suggests that AVH are not related to regional brain dysfunction alone, but rather to abnormal neural network coupling in several distinct neural networks including systems engaged in language, attention, executive function, memory and self-referential processing (Stephane et al., 2001; Allen et al., 2008; Wolf et al., 2011; Diederen et al., 2013). Thus, single-site stimulation may not fully cover all key regions involved in AVH pathophysiology. In this regard, bilateral or bifocal stimulation could be a promising approach. Based on the hypothesis of temporal hyperactivity and frontal hypoactivation in schizophrenia patients presenting with AVH, Brunelin and colleagues used a different non-invasive stimulation method, i.e., tDCS (Brunelin et al., 2012). Unlike TMS, in tDCS a weak direct current passes through the brain between two electrodes, i.e., modulation of two spatially remote regions is possible. Brunelin and co-workers used cathodal left temporoparietal junction (TPJ) stimulation and anodal left dorsolateral prefrontal stimulation. After five days of treatment a significant decrease of hallucinatory symptoms was shown, and this effect remained significant three months after stimulation. These findings were recently replicated (Mondino et al., 2015) and provides a promising outlook for further clinical trials. Nevertheless, given that tDCS is a relatively new technique employed in AVH treatment, several stimulation parameters (e.g., electrode placement and stimulation intensity, frequency and duration) have to be investigated in more detail to optimize future treatment options (Koops et al., 2015).

It is noteworthy that although the lateral prefrontal and temporal cortices clearly are involved in AVH generation, there is also good evidence suggesting a role of cortical midline regions in AVH symptom expression. Abnormal cerebral blood flow could also be detected not only in the primary temporal cortex and Broca’s area, but also in the cingulate cortex (Wolf et al., 2012; Kindler et al., 2013). In a recent study exploring resting-state functional connectivity of the brain, cross-network abnormalities could be detected between the so called “default mode network” (DMN) and the “salience network,” including core midline regions such as the bilateral paracingulate cortex and bilateral anterior cingulate cortex (Alonso-Solís et al., 2015). Of note, DMN subsystems have been essentially involved in self-referential and mnemonic processes (Andrews-Hanna et al., 2010; Sambataro et al., 2013). Abnormal network interactions between the DMN and language-processing and auditory networks could well explain deficient self-monitoring and a lack of self-referential attribution of voices (Northoff and Qin, 2011). This body of evidence indicates important contributions of cortical midline regions to the pathomechanisms of persistent AVH. TMS alone might be insufficient to stimulate these regions in treatment-resistant patients.

### The Challenge of Phenotypic Diversity

When treating AVH in patients with schizophrenia using focal stimulation techniques, the clinical endpoint appears to be clearly defined. In the vast majority of cases, this is at least a reduction in overall AVH severity. Yet it should be kept in mind that schizophrenia is a phenomenologically heterogeneous disorder with several distinct phenotypic presentations at both the clinical and neurobiological level, and the very same heterogeneity also applies to persons with chronic AVH. In addition, the multidimensionality of AVH has been long acknowledged by phenomenological research (Kronmüller et al., 2011; McCarthy-Jones et al., 2014), but research has only recently begun to specifically explore therapeutic effects on distinct symptom domains (Leff et al., 2013).

Apart from refining and technically developing stimulation techniques *per se*, a major focus of future research will be the identification of markers which can predict stimulation treatment response. An approach which might prove to be helpful for predicting responders and non-responders in the future is subtyping AVH patients according to both neurobiological and clinical criteria. For instance, it has been attempted, albeit with limited success, to improve responsiveness to rTMS by targeting the site of maximal neural activation associated with the hallucinatory event (Slotema et al., 2011). More recently, Homan and colleagues (Homan et al., 2012) showed that higher resting-brain perfusion as measured with arterial spin labeling in the left STC prior to treatment predicted a clinical response to rTMS (Homan et al., 2012). This marker may guide stratification strategies in future interventional trials. Also, it is important to acknowledge that certain symptom characteristics, such as location of voices in inner our outer space, may map to distinct neural correlates. In this respect, a relationship between white matter volume in the right temporal junction and spatial features such as outer vs. inner location of voices has been identified (Plaze et al., 2011). However, stimulation of the right temporal lobe could not show a superior treatment effect. A possible explanation is that there may well be structural differences between hallucinating characterized by “physical” features with yet unknown consequences for brain function and treatment response. Another explanation for non-response to stimulation could include neural ceiling effects, e.g., related to various degrees of subjective symptom control. Over time, patients with persistent AVH seek for ways of coping with their voices, e.g., by deliberately directing their attentional focus to specific external stimuli (which can be auditory) or by employing individual modes of verbal control. The degree of control over AVH is associated with distinct frontotemporal cortical correlates in contrast to physical or affective symptom dimensions (Wolf et al., 2012). Also, increased frontotemporal connectivity in hallucinating patients is modulated by the degree of control over verbal material (Lavigne et al., 2015). Thus, it is possible that different degrees of control over AVH severity prior to therapeutic stimulation could influence treatment response.

McCarthy-Jones and colleagues proposed different subtypes of AVH, which might respond to different treatment modalities. These subtypes may be identified at the levels phenomenology, cognition, neurology, etiology, treatment response, diagnosis, and voice hearer’s own interpretation (McCarthy-Jones et al., 2014). Particularly, an AVH subtype characterized at a neural level by chronic deafferentiation of the auditory cortex is proposed, according to the hypothesis of AVH as misattributed forms of inner speech (Ford and Mathalon, 2005). This subtype might be specifically responsive to focal stimulation treatment, i.e., rTMS or tDCS. Furthermore, specific subtypes might show both common and distinct regions of activation in both “symptom capture” and “symptom interference” studies, so that future neuroimaging studies may consider specific subtypes in their protocol and report details of AVH phenomenology. The majority of functional neuroimaging studies used total severity and frequency scores of hallucinations (Auditory Hallucinations Rating Scale (AHRS), Auditory Hallucinations Subscale/Psychotic Symptom Rating Scale [AHS/PSYRATS]) as main outcome parameters. To discriminate more fine-grained aspects of change in hallucinations, especially in homogenous subgroups, it might be advantageous to describe different phenomenological dimensions before and their changes after focal therapy. The PSYRATS and a 4-dimensional model within the AHS has previous been recommended to integrate into research and clinical applications (Woodward et al., 2014).

## Conclusion

In the past decade non-invasive brain stimulation techniques became increasingly relevant for the treatment of drug-refractory AVH. Evidence for ECT for specifically treating AVH is very limited. There is evidence for beneficial effects of rTMS over left temporal and temporoparietal areas, but effect sizes for this treatment modality are moderate, and beneficial long-term effects are unlikely. It has been suggested that rTMS may reduce aberrant internally generated activity associated with AVH at the site of stimulation. Still, the role of rTMS in changing aberrant network function putatively involved in the generation of AVH has to be clarified. A further major challenge for future research is identifying of patients who do respond to treatment and those who do not or only insufficient. Supported by neuroimaging evidence, the magnitude of left STC activity has been promoted as a potential predictor of clinical improvement. Given the phenomenological diversity of schizophrenia and AVH in particular, it is expected that subtyping patients with AVH will essentially contribute to identify responders from non-responders for focal augmentative therapies. In this respect, in accordance with other authors we strongly advocate further development of reliable and valid psychometric assessments and neurobiological markers paralleling the optimization of future stimulation protocols. Other neuromodulatory interventions, such as tDCS provide very promising data as well but larger trials are needed.

## Conflict of Interest Statement

The authors declare that the research was conducted in the absence of any commercial or financial relationships that could be construed as a potential conflict of interest.
